# Upregulation of fibronectin following loss of p53 function is a poor prognostic factor in ovarian carcinoma with a unique immunophenotype

**DOI:** 10.1186/s12964-020-00580-3

**Published:** 2020-07-07

**Authors:** Ako Yokoi, Toshihide Matsumoto, Yasuko Oguri, Yoshinori Hasegawa, Masataka Tochimoto, Mayu Nakagawa, Makoto Saegusa

**Affiliations:** 1 Department of Pathology, Kitasato University School of Medicine, 1-15-1 Kitasato, Minami-ku, Sagamihara, Kanagawa 252-0374 Japan; 2 Department of Applied Genomics, Kazusa DNA Research Institute, Laboratory of Clinical Omics Research, 2-6-7 Kazusakamatari, Kisaratsu, Chiba, 292-0818 Japan

**Keywords:** Ovarian carcinoma, p53, HNF-1β, ARID1A, Fibronectin, Prognosis, Immunophenotype, Cell proliferation, Cell mobility, Apoptosis

## Abstract

**Background:**

We previously demonstrated that ovarian high grade serous carcinomas (OHGSeCa) and ovarian clear cell carcinomas (OCCCa) with an HNF-1β+/p53+/ARID1A+ immunophenotype were associated with the worst unfavorable prognosis. To clarify the molecular mechanisms underlying this finding, we focused on alterations in the p53 signaling pathway in these tumors.

**Methods:**

Changes in cell phenotype and function following knockdown of wild-type p53 (p53-KD) were assessed using OCCCa cells expressing endogenous HNF-1β and ARID1A. The prognostic significance of molecules that were deregulated following p53-KD was also examined using 129 OCCCa/OHGSeCa cases.

**Results:**

p53-KD cells had increased expression of Snail, phospho-Akt (pAkt), and pGSK3β, and decreased E-cadherin expression, leading to epithelial-mesenchymal transition (EMT)/cancer stem cell (CSC) features. The cells also exhibited acceleration of cell motility and inhibition of cell proliferation and apoptosis. Next generation sequencing revealed that fibronectin (FN) expression was significantly increased in the p53 KD-cells, in line with our observation that wild-type p53 (but not mutant p53) repressed *FN1* promoter activity. In addition, treatment of OCCCa cells with FN significantly increased cell migration capacity and decreased cell proliferation rate, independent of induction of EMT features. In clinical samples, FN/p53 scores were significantly higher in OCCCa/OHGSeCa with the HNF-1β+/p53+/ARID1A+ immunophenotype when compared to others. Moreover, high FN/high p53 expression was associated with the worst overall survival and progression-free survival in OCCCa/OHGSeCa patients.

**Conclusion:**

These findings suggest that upregulation of FN following loss of p53 function may impact the biological behavior of OCCCa/OHGSeCa, particularly in tumors with an HNF-1β+/p53+/ARID1A+ immunophenotype, through alterations in cell mobility and cell proliferation. The accompanying induction of EMT/CSC properties and inhibition of apoptosis due to p53 abnormalities also contribute to the establishment and maintenance of tumor phenotypic characteristics.

Video Abstract

## Background

**1**Ovarian epithelial carcinomas (OECa) are among the most aggressive tumors and the leading cause of mortality among all types of malignancies in the female reproductive system [1]. Since the ovaries have a relatively inaccessible location and ovarian carcinoma patients very often lack symptoms in the early neoplastic stage, more than 75% of the patients are diagnosed with advanced stage disease that is characterized by metastasis to the peritoneal cavity [2]. In addition, approximately 80% of advanced stage patients have residual disease after surgery and receive front-line platinum-based combination chemotherapy; these individuals have a median progression-free survival (PFS) of 18 months [3].

p53 is widely acknowledged as the most frequently mutated gene in human malignancy and its mutational status is a prognostic marker in several tumor types [4]. Activated wild-type p53 (p53wt) acts as a checkpoint control for recognizing damaged DNA, allowing DNA repair and delayed entrance into the DNA replication phase of the cell cycle; together with observations that the incidence of tumorigenesis increases in p53 null or mutant tissues, these data confirm that p53 is a bona fide tumor suppressor [5]. Mutations in the *TP53* gene are found in more than 50% of human malignancies and its inactivation can occur at various stages depending on the tissue that gives rise to the tumor. Therefore, loss of p53 function can promote neoplastic transformation as well as progression of established tumors to a more aggressive disease stage [6, 7]. In OECa, and particularly in ovarian high-grade serous carcinomas (OHGSeCa), mutant p53 (p53mt) missense mutations are frequently found in the hotspot codon R175, R248, and R273 (http://www-p53.iarc.fr/) that are critical contact residues in the p53 DNA-binding domain. The mutations occur early during tumorigenesis, most likely in precursor lesions of OECa, highlighting the importance of p53mt as a driver of the malignancy [8–11].

We previously developed an effective immunoprofiling classification system for OECa using only 4 immunohistochemical markers (HNF-1β, p53, ARID1A, and WT1) [12]. Using this system, we demonstrated that tumors with an HNF-1β+/p53+/ARID1A+ immunophenotype including OHGSeCa and ovarian clear cell carcinomas (OCCCa) were associated with the most unfavorable prognosis. In this study, we hypothesized that alterations in the p53 signaling pathway may play a key role in determining phenotypic characteristics in OECa with the HNF-1β+/p53+/ARID1A+ immunophenotype. To test this, we set out to first examine the effects of knocking down p53wt (p53-KD) in OCCCa cells expressing endogenous HNF-1β and ARID1A. Next, we applied a next generation sequencing (NGS) assay to identify the molecules associated with loss of p53 function. Finally, we examined associations between molecules that were differentially expressed following p53-KD, tumor phenotypic characteristics and prognostic significance in OHGSeCa and OCCCa.

## Methods

### Plasmids and cell lines

The p53-specific short hairpin RNA (shRNA) oligonucleotides were designed as described previously [13]. Single-stranded p53 oligonucleotides were annealed and then cloned into *BamH*1-*EcoR*V sites of RNAi-Ready pSIREN-RetroQ vector (Takara, Shiga, Japan), according to the manufacturer’s instructions. The p53mt (R248Q) was generated by PCR-based methods using a pCMV-p53wt construct. The human *Fibronectin 1* promoter (UCSC genome browser, https://genome.ucsc.edu/) between − 2028 and − 23 (where + 1 represents the transcription start site) was also generated by PCR and was cloned into the pGL3B vector (Promega, Madison, WT, USA). The primer sequences for the PCR reaction used in this study are listed in Table 1. pCMV-p53wt, pGL3B-(− 1109/+ 36) Snail luc, pGL3B-(− 899/+ 47) HNF-1β luc, and pGL3B-(− 140/+ 216) HNF-1β luc were also used as described previously [14–16].

**Table 1:** Primer sequences used in this study

Assay	Gene		Sequence	
shRNA	p53 (position 775 to 794)	Forward	5′-GATCCGGACTCCAGTGGTAATCTACTTTCAAGAGAAGTAGATTACCACTGGAGTCTTTTTTG-3’	
		Reverse	5′-AATTCAAAAAAGACTCCAGTGGTAATCTACTTCTCTTGAAAGTAGATTACCACTGGAGTCCG-3’	
Mutagenesis	p53 mutant type (R248Q)	Forward	5′-ATGAACCaGAGGCCCATCCTCACCATCATCA-3’	
		Reverse	5′-GGGCCTCtGGTTCATGCCGCCCATGCA-3’	
mRNA	Fibronectin 1	Forward	5′- CCATCGCAAACCGCTGCCAT-3’	
		Reverse	5′-AACACTTCTCAGCTATGGGCTT-3’	
Promoter	Fibronectin 1 (−2028 to − 23)	Forward	5′-CGGCTAGCTTCAGTGCAGTAAATATATC-3’	
		Reverse	5′- ATCTCGAGTTATATGGGACGGTCCCCTCCCGCC-3’	

Four OCCCa cell lines, OVISE, ES2, OVTOKO, and TOV-21G were used as described previously [13, 16, 17], and two OHGSeCa cell lines, OVSAHO and OVCAR-3, were obtained from the National Institute of Biomedical Innovation (Osaka, Japan) and the American Type Culture Collection (Manassas, VA, USA), respectively. p53 shRNA knockdown cells were established using OVISE cells, which have a wild-type *p53* gene and abundant expression of endogenous HNF-1β and ARID1A (Supplementary Figure S1), as described previously [13, 17]. In addition, spindle-shaped cells were defined as those that showed narrow and elongated phenotypes, along with weak or absent adhesions between cells, as described previously [17].

### Antibodies and reagents

Anti-p53, anti-p21^waf1^, anti-cyclin D1, and anti-bcl2 antibodies were purchased from Dako (Copenhagen, Denmark). Anti-HNF-1β, anti-GSK-3β, anti-Rb, anti-p27^kip1^, anti-XIAP, anti-bax, and anti-integrin β1 antibodies were obtained from BD Biosciences (San Jose, CA, USA). Anti-ARID1A, anti-cyclin B1, and anti-MDM2 antibodies were from Santa Cruz Biotechnology (Santa Cruz, CA, USA). Anti-Snail, anti-Akt, anti-phospho(p) Akt Serine473, anti-pGSK-3β Serine9, anti-pRb Serine807/811, anti-cleaved caspase-3, and anti-integrin β3 antibodies were from Cell Signaling Technology (Danvers, MA, USA). Anti-fibronectin (FN), anti-E-cadherin, and anti-β-actin antibodies were obtained from Abcam (Cambridge, MA, USA), Takara (Shiga, Japan), and Sigma-Aldrich Chemicals (St. Louis, MO, USA), respectively. Anti-cyclin A2 and anti-integrin β2 antibodies were from Novocastra (Newcastle, UK) and Merck KGaA (Darmstadt, Germany), respectively. FN (catalog number #F2006) and cisplatin (CDDP: #479306) were purchased from Sigma-Aldrich Chemicals.

### Transfection

Transfection was carried out using LipofectAMINE PLUS (Invitrogen, Carlsbad, CA, USA) as described previously [14–16]. Luciferase activity was assayed as described previously [14–16].

### Reverse transcription (RT)-PCR

cDNA was synthesized from 2 μg of total RNA. Amplification by RT-PCR was carried out in the exponential phase to allow comparison among cDNA synthesized from identical reactions using specific primers (Table 1). Primers for the *HNF-1β*, *Snail*, and *GAPDH* genes were also applied, as described previously [14–16]. The signal intensity was analyzed by ImageJ software version 1.41 (NIH, Bethesda, MD, USA).

For quantitative analysis, real-time RT-PCR was also conducted using a Power SYBR Green PCR Master Mix (Applied Biosystems, Foster City, CA, USA). Fluorescent signals were detected using the ABI 7500 Real-time PCR System SDS Software (Applied Biosystems).

### Western blot assay

Total cellular proteins were isolated using RIPA buffer [20 mM Tris-HCl (pH 7.2), 1% Nonidet P-40, 0.5% sodium deoxycholate, 0.1% sodium dodecyl sulfate]. Aliquots of the proteins were resolved by SDS-PAGE, transferred to membranes, and probed with primary antibodies, coupled with the ECL detection system (Amersham Pharmacia Biotechnology, Tokyo, Japan).

### Flow cytometry and Aldefluor assay

Cells were fixed using 70% alcohol and stained with propidium iodide (Sigma) for cell cycle analysis. Aldehyde dehydrogenase 1 (ALDH1) enzyme activity in viable cells was determined using a fluorogenic dye-based Aldefluor assay (Stem Cell Technologies, Grenoble, France) according to the manufacturer’s instructions. The prepared cells were analyzed by flow cytometry using BD FACS Calibur (BD Biosciences) and CellQuest Pro software version 3.3 (BD Biosciences).

### Cell counting Kit-8 assay

The quantitation of viable cell number in proliferation after CDDP treatment was carried out using a Cell Counting Kit-8 (CCK-8; Dojindo Lab, Kumamoto, Japan), according to the manufacturer’s instructions.

### Wound healing assay

Cells were seeded into 24-well tissue culture plates, and grown to reach almost total confluence. After a cell monolayer formed, a wound was scratched with a sterile 200-μl tip. The area of the wound was analyzed by ImageJ software version 1.41 (NIH). Cell migration parameters were calculated in pixels as wound closure.

### Migration assay

Cell migration was determined using 24-well Transwell chambers with an 8-μm pore size (Corning, NY, USA). The lower chamber was filled with medium containing 10% serum. Cell were suspended in serum-free medium with or without FN and transferred into the upper chamber. After 24 h, the number of cells stained by hematoxylin-eosin (HE) on the bottom surface of the polycarbonate membranes was counted visually using a light microscope.

### Apoptotic index

Apoptotic cells were identified in HE-stained sections, according to the criteria of Kerr et al. [18]. A total 10 fields were randomly selected, and the number of apoptotic cells was calculated by counting the mean number of apoptotic figures per high power field (HPF).

### NGS assay

Total RNAs were extracted from OV-shp53 and mock cells using the NucleoSpin RNA system (Takara). The concentration and quality of the RNA was verified with the Quantus Fluorometer (Promega) and Agilent 2100 Bioanalyzer, respectively. All the samples showed RIN values over 9. Total RNA (500 ng) was used for RNA library preparation, according to the instructions of the Quant Seq 3′ mRNA-seq library preparation kit FWD from Illumina (Lexogen, Vienna, Austria). The libraries were PCR-amplified for 12 cycles.

Sequencing of the libraries (via single-end 75-bp reads) was conducted on the Illumina NextSeq500 system. All data analyses were conducted using Strand NGS (v3.2, Agilent Technologies). The adapter sequences were removed from the raw reads, and base trimming was performed from the 3′ end of each read to remove bases with quality below Q10 up to a minimum length of 25 bp. Each read was mapped to the reference human genome hg38 with default settings. Expression patterns of transcripts were compared after normalization of DESeq [19] using default settings.

### TCGA data analysis

The Cancer Genome Atlas (TCGA) OHGSeCa annotated *TP53* gene alteration and mRNA expression data (RNA Seq V2 PSEM) for HNF-1β and ARID1A were extracted from cBioportal for Cancer Genomics (http://www.cbioportal.org/) for 398 OHGSeCa cases.

### Clinical cases

A total of 199 cases of OECa, surgically resected at Kitasato University Hospital between 2006 and 2017, were selected from our patient records according to the criteria of the 2014 World Health Organization classification [20]. All patients underwent oophorectomy with or without hysterectomy. None of the patients had received chemotherapy or any other preoperative treatment, while most patients had received Paclitaxel/Carboplatin-based chemotherapy after surgical treatment. Of these, 99 cases including 41 OHGSeCa and 58 OCCCa showed complete resection of the tumors, while 28 cases including 17 OHGSeCa and 11 OCCCa had residual tumors after debulking surgery.

Evaluation of relapse and disease progression was conducted on the basis of radiologic image findings. The tumor cases investigated were comprised of 58 OHGSeCa, 9 ovarian low grade serous carcinoma, 29 ovarian endometroid carcinomas, 71 OCCCa, and 30 ovarian mucinous carcinomas. All tissues were routinely fixed in 10% formalin and processed for embedding in paraffin wax. Approval for this study was given by the Ethics Committee of the Kitasato University School of Medicine (B16–10).

### Immunohistochemistry (IHC)

IHC was performed using a combination of the microwave-oven heating and polymer immunocomplex (Envision, Dako) methods, as described previously [14–16].

For evaluation of IHC findings, scoring of nuclear/cytoplasmic immunoreactivity was performed, on the basis of the percentage of immunopositive cells and the immunointensity, with multiplication of values of the two parameters, as described previously [14–16]. In addition, FN/p53 score was generated by multiplication of the values of the two scores.

To evaluate the prognostic significance of FN and p53 expression, the scores were divided into two categories (high and low) with the mean values as the cut-off in each category (Table 2). With regard to p53, cases that were completely negative for p53 immunoreactivity were categorized into the high p53 score group (score = 12), since combining 2 IHC labeling patterns associated with p53 mutations (0% and 60–100% positive cells) correctly identified a mutation in 94% of cases [21].

**Table 2:** Association between clinicopathological factors and Fibronectin and p53 IHC scores in OCCCa/OHGSeCa

			Fibronectin IHCscore			p53 IHCscore		
			High (≧3)	Low (3<)	*p*-value	High (≧6)	Low (6<)	p-value
		n	n (%)	n (%)		n (%)	n (%)	
Age	<58	61	14 (23.0)	47 (77.0)	0.7	23 (37.7)	38 (62.3)	0.6
(years)	≧58	66	12 (18.2)	54 (81.8)		29 (44.0)	37 (56.0)	
Clinical	I	40	7 (17.5)	33 (82.5)	0.7	4 (10.0)	36 (90.0)	< 0.0001
FIGO stage	II / III / IV	87	19 (21.8)	68 (78.2)		48 (55.2)	39 (44.8)	
Histology	OCCCa	69	20 (29.0)	49 (71.0)	0.01	6 (8.7)	63 (91.3)	< 0.0001
	OHGSeCa	58	6 (10.3)	52 (89.7)		46 (79.3)	12 (20.7)	
Tumor size	<11.2	82	11 (13.4)	71 (86.6)	0.01	41 (50.0)	41 (50.0)	0.008
(cm)	≧11.2	45	15 (33.3)	30 (66.7)		11 (24.4)	34 (75.6)	
LN	Positive	27	11 (40.7)	16 (59.3)	0.008	15 (55.6)	12 (44.4)	0.1
metastasis	Negative	100	15 (15.0)	85 (85.0)		37 (37.0)	63 (63.0)	
Distant	Positive	12	4 (33.3)	8 (66.7)	0.4	10 (83.3)	2 (16.7)	0.004
metastasis	Negative	115	22 (19.1)	93 (66.7)		42 (36.5)	73 (63.5)	

*LN* lymph node, *n* number of cases

### Statistics

Comparative data were analyzed using the Mann-Whitney *U*-test. Overall survival (OS) was calculated as the time between onset and death or the date of the last follow-up evaluation. PFS was also examined from the onset of treatment until relapse, disease progression, or last follow-up evaluation. OS and PFS were estimated using the Kaplan-Meier methods, and the statistical comparisons were made using the log rank test. Univariate and multivariate analyses were performed using the Cox proportional hazards regression model. The cut-off for statistical significance was set as *p* < 0.05.

## Results

### Loss of p53 function leads to induction of EMT features

**Fig. 1:**
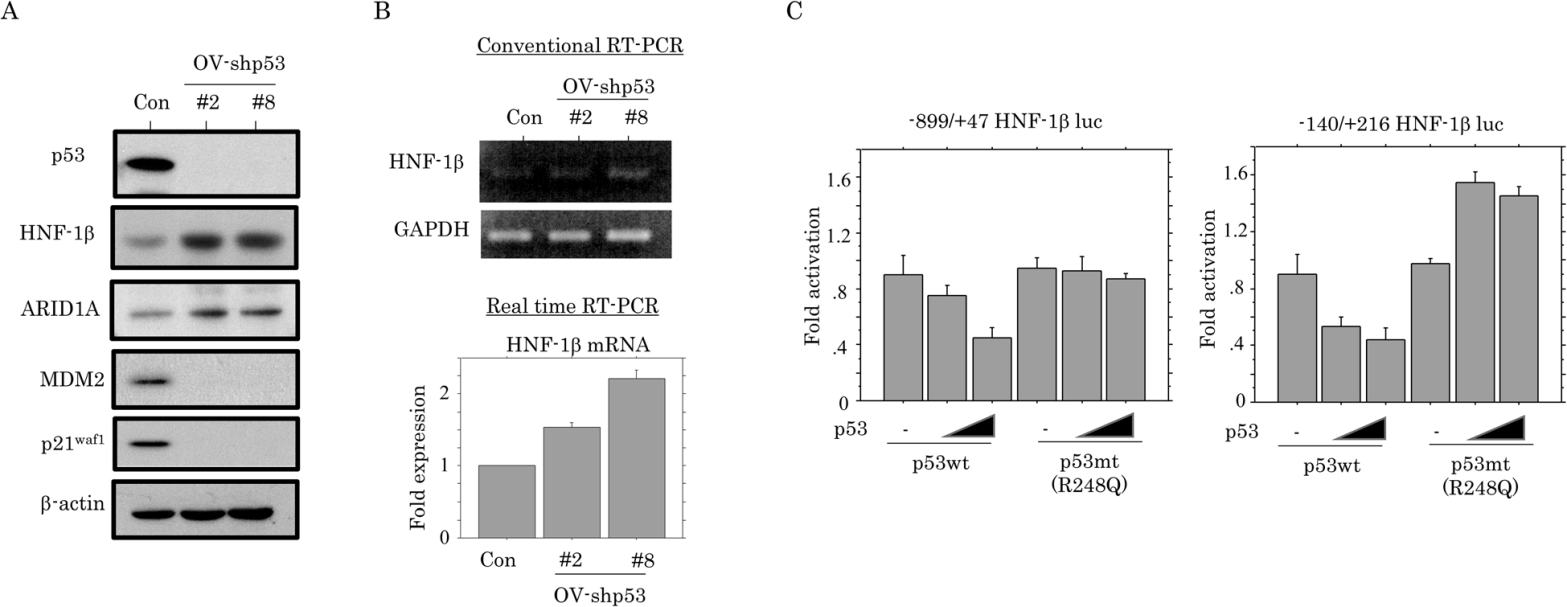
Expression of HNF-1β and ARID1A in p53-KD cells. **a** Western blot analysis for the indicated proteins in total lysates from OV-shp53 and control cells (Con). **b** Analysis of endogenous *HNF-1β* mRNA expression by conventional (upper) and real time RT-PCR assay (lower) for OV-shp53 and control cells (Con). The fold changes in mRNA expression detected by real time RT-PCR are presented as means±SDs. The experiment was performed in triplicate. **c** OVISE cells were transfected with two *HNF-1β* reporter constructs, respectively, together with either p53wt or p53mt. Relative activity was determined based on arbitrary luciferase light units normalized to pRL-TK activity. The activities of the reporter plus the effector relative to that of the reporter plus empty vector are shown as means±SDs. The experiment was performed in duplicate

To examine the phenotypic characteristics of OECa cells with the HNF-1β+/p53+/ARID1A+ immunophenotype, we first established two independent cell lines in which p53 expression was blocked by a p53-specific shRNA (OV-shp53#2 and #8) using OVISE (OV) cells. The OV-p53-KD cells showed increased expression of both HNF-1β and ARID1A, in contrast to decreased expression of MDM2 and p21^waf1^, which are p53 target genes (Fig. 1a). *HNF-1β* mRNA expression was also increased in the OV-p53-KD cells as compared to the mock cells (Fig. 1b), in line with the observation of dose-dependent repression of *HNF-1β* promoter activity following transfection of p53wt. In contrast, the repressive effects were not evident when p53mt was transfected (Fig. 1c).

**Fig. 2:**
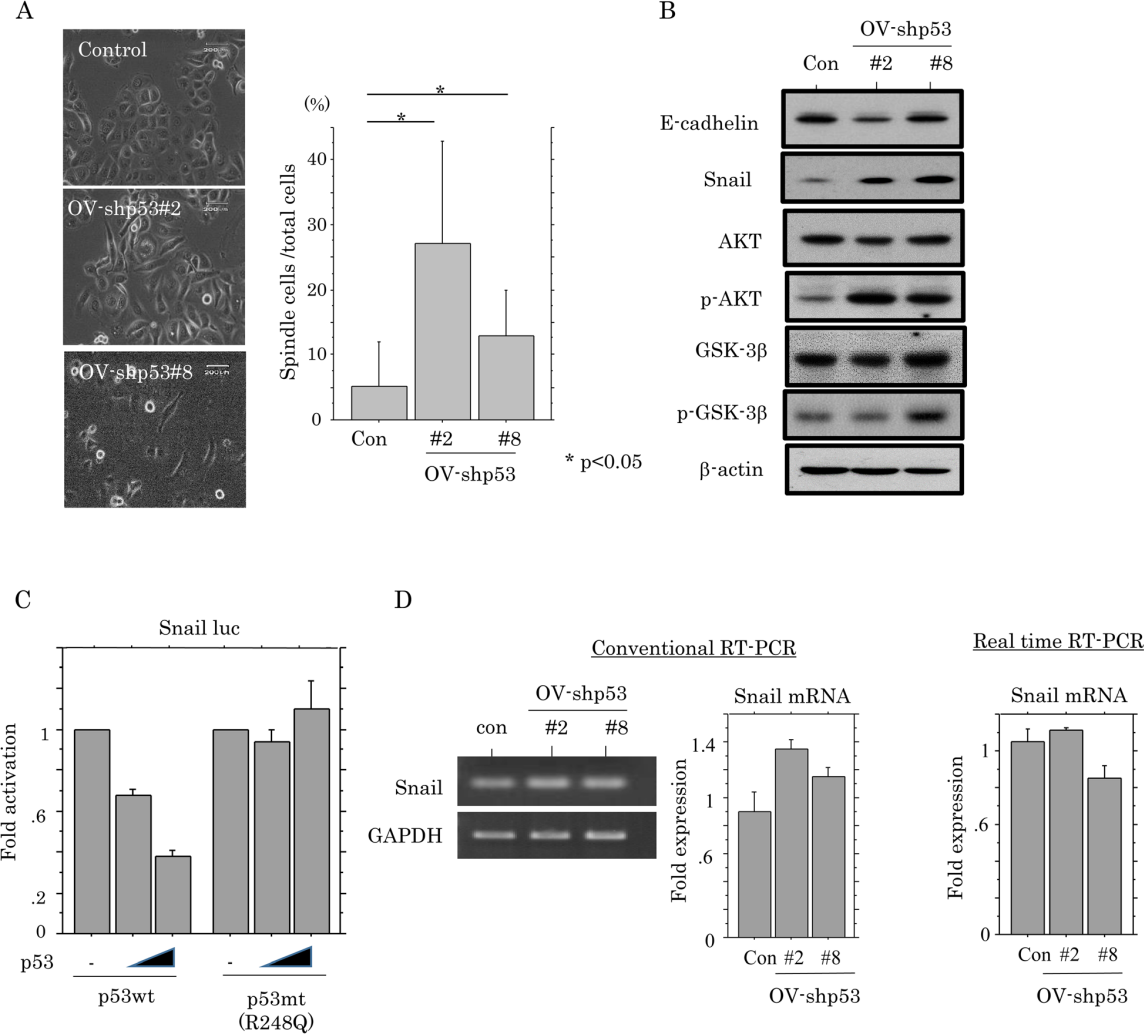
Relationship between cell phenotypic characteristics and p53-KD. **a** Left: phase contrast images of OV-shp53 cells. Note the changes in cell morphology toward fibroblastic appearances in OV-shp53 cells. Right: the numbers of spindle-shaped cells are presented as means±SDs. **b** Western blot analysis for the indicated proteins in total lysates from OV-shp53 and control cells (Con). **c** OVISE cells were transfected with Snail reporter constructs, together with either p53wt or p53mt. Relative activity was determined based on arbitrary luciferase light units normalized to pRL-TK activity. The activities of the reporter plus the effector relative to that of the reporter plus empty vector are shown as means±SDs. The experiment was performed in duplicate. **d** Analysis of endogenous *Snail* mRNA expression by conventional (left) and real time RT-PCR assay (right) for OV-shp53 and control cells (Con). The signals of endogenous *Snail* mRNA expression in the conventional RT-PCR assay were normalized to *GAPDH*. The fold changes in mRNA expression detected by both assays are presented as means±SDs. The experiment was performed in triplicate

The OV-p53-KD cells also demonstrated a significant switch towards a fibroblastic morphology (Fig. 2a), along with increased expression of Snail, pAkt, and pGSK-3β, and decreased E-cadherin expression (Fig. 2b). Although *Snail* promoter activity was inhibited by p53wt, but not p53mt (Fig. 2c), changes in mRNA expression were relatively minor in OV-p53-KD cells as compared to the mock cells (Fig. 2d). These findings suggest that loss of p53 function contributes to increased expression of HNF-1β and ARID1A, leading to induction of epithelial-mesenchymal transition (EMT) features, probably through post-translational regulation of Snail expression.

### Loss of p53 function is associated with CSC features and acceleration of cell mobility

**Fig. 3:**
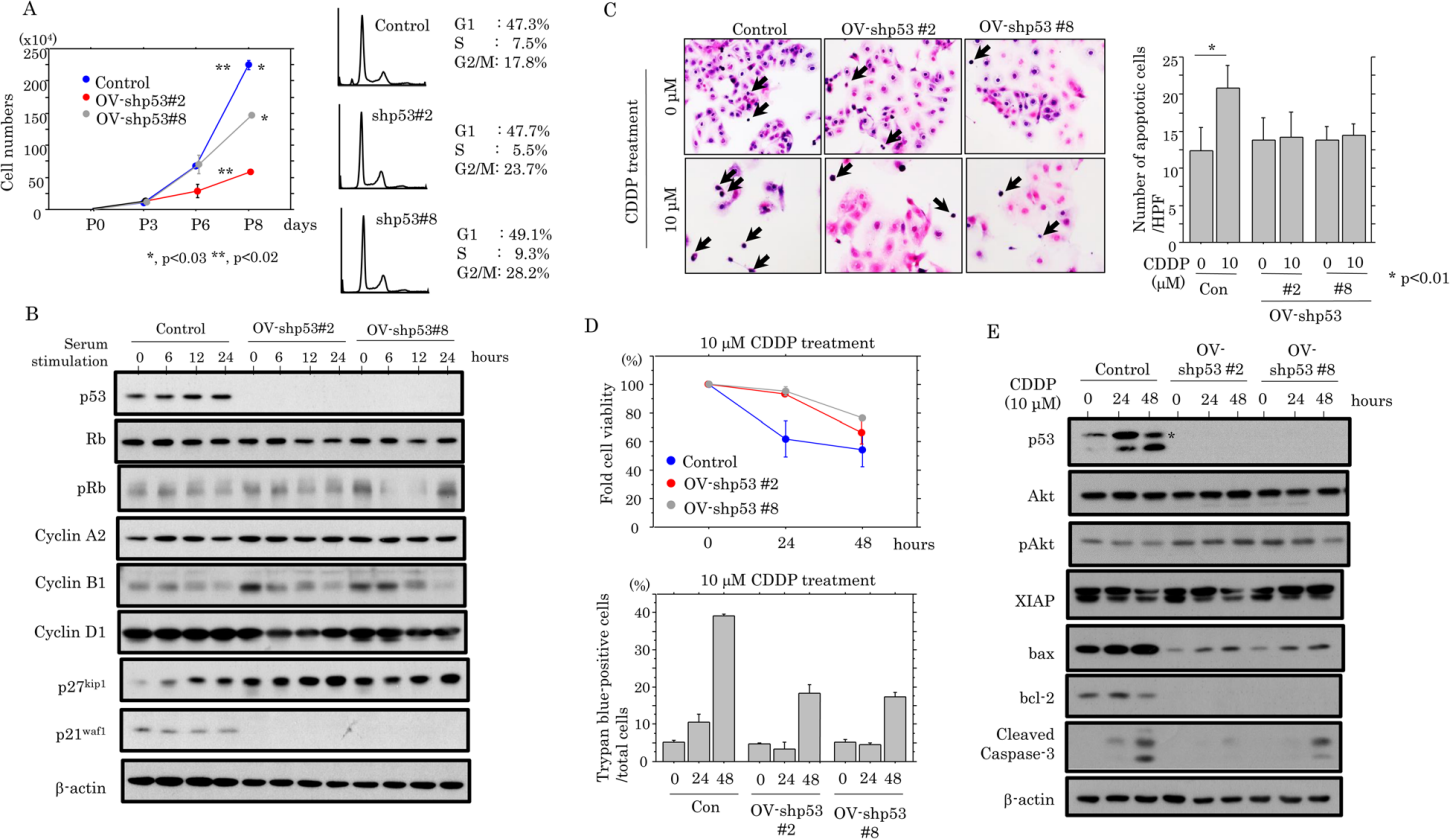
Relationship between p53-KD and cell proliferation or apoptosis. **a** Left: two independent OV-shp53 and control cell lines were seeded at low density. The cell numbers are presented as means±SDs. P0, P3, P6, and P8 are 0, 3, 6, and 8 days after seeding, respectively. Right: FACS analysis of OV-shp53 and control cells at 3 days after seeding (P3). **b** Western blot analysis for the indicated proteins in total lysates from OV-shp53 and the mock cells. **c** Left: after treatment with 10 μM CDDP, OV-shp53 and control cells undergoing apoptosis are indicated by arrows. Original magnification, × 400. Right: the numbers of apoptotic cells are demonstrated as means±SDs. Con, control. d Upper: treatment of OV-shp53 and control cells with 10 μM CDDP for the times shown. Cell viability was measured using the CCK-8 kit. The viability in the absence of CDDP treatment (0 h) is set as 100%. Lower: treatment of OV-shp53 and control cells with 10 μM CDDP for the times shown. The numbers of trypan blue-positive cells (non-viable cells) are presented as mean ± SD. This experiment was performed in triplicate using independent samples. **e** Western blot analysis for the indicated proteins in total lysates from OV-shp53 and control cells treated with 10 μM CDDP

To examine whether p53-KD affects cell proliferation, the two independent OV-p53-KD cell lines were seeded at low density. OV-p53-KD cells tended to proliferate more slowly, particularly in the exponential growth phase, along with an increased proportion of cells in G2/M phase of the cell cycle (Fig. 3a). To further examine alterations in expression of several cell cycle-related molecules during cell growth, the OV-p53-KD cells were rendered quiescent by serum starvation and were subsequently stimulated with serum. At 6, 12, and 24 h after release into the cell cycle, p27^kip1^ expression was substantially increased in OV-p53-KD cells relative to the mock cells, in contrast to the progressive reduction of cyclin B1 expression in the former (Fig. 3b).

Next, we examined the association between loss of p53 function and apoptotic features in response to cytotoxic effects. Treatment of OV-p53-KD cells with CDDP showed decreased apoptotic cells as compared to the mock (Fig. 3c), in line with the results of increased cell viability during CDDP treatment (Fig. 3d). The expression of cleaved caspase-3, as well as bax and bcl-2, were also apparently decreased in the OV-p53-KD cells as compared to mock cells, in contrast to increased pAkt, but not XIAP, expression (Fig. 3e).

**Fig. 4:**
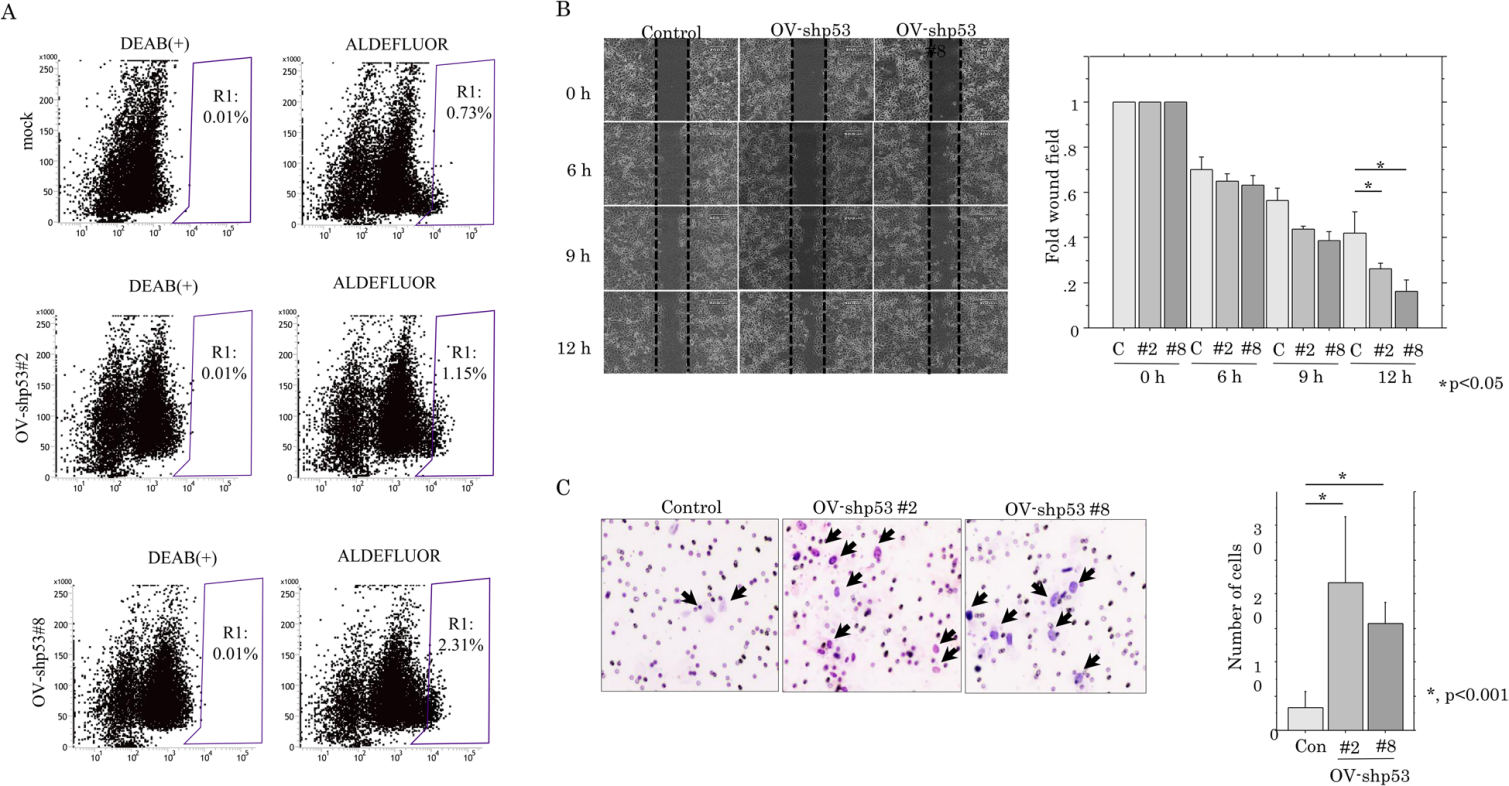
Relationship between p53-KD and cancer stem cell features or cell migration. **a** Aldefluor analysis in OV-shp53 and control cells. Note the R1 populations including the ALDH^high^ population with cancer stem cell-like features. **b** Left: wound-healing assay with OV-shp53 and control cells. A scratch ‘wound’ was introduced to the middle of wells containing cell growth to confluency, and phase contrast images were taken after 6, 9, and 12 h. Right: the values of wound areas in 0 h were set as 1. The fold wound areas are presented as means±SDs. C, control. **c** Migration rate measured using transwell assay. Left: the OV-shp53 and control cells were seeded in a 24-well transwell plates and incubated for 24 h in medium without serum. Cells were stained with HE and counted using a light microscope. Right: the numbers of migrated cells are presented as means±SDs

Since EMT promotes stem cell properties and further generates cells with cancer stem cell (CSC)-like features [22], we examined the association between loss of p53 function and CSC properties. As shown in Fig. 4a, there was a significant increase in the ALDH^high^ population, which includes a high percentage of CSC-like cells, in the p53-KD cells compared to the mock cells.

To further examine whether loss of p53 function contributes to cell motility, we carried out scratch and migration assays. The OV-p53-KD cells refilled wounded empty spaces more rapidly (Fig. 4b), in line with the significantly increased migration rates as compared to the mock cells (Fig. 4c). These findings suggest that loss of p53 function also engenders CSC features and accelerates cell motility in OVISE cells; these changes are accompanied by inhibition of cell proliferation and susceptibility to apoptosis.

### Upregulation of FN expression by loss of p53 function

**Fig. 5:**
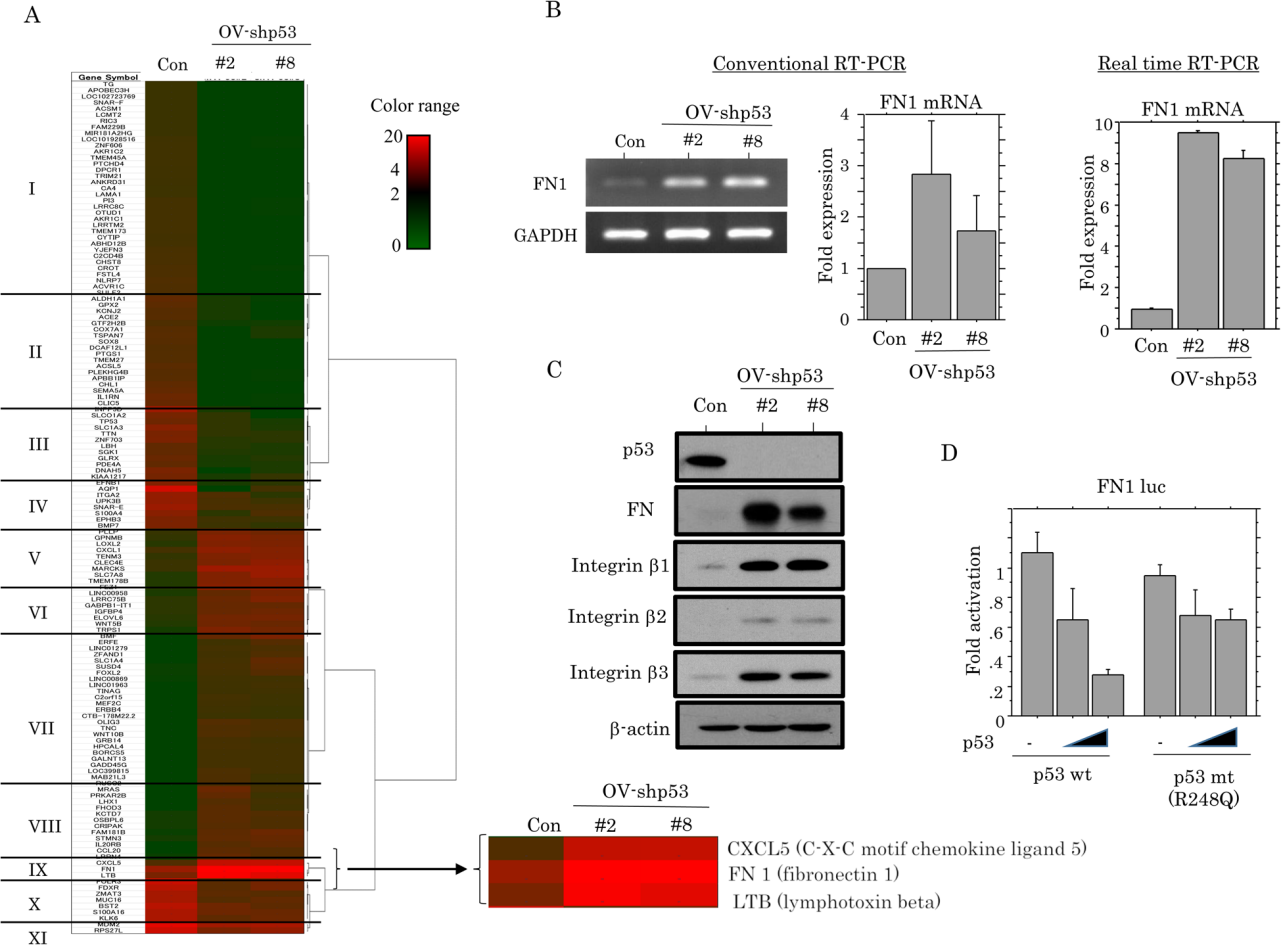
Relationship between p53 and FN expression. **a** Unsupervised hierarchical clustering of mRNA expression detected by next generation sequencing in OV-shp53 and control cells (Con). The expression level of each mRNA is colored; red, black, and green indicated high (> 4), neutral (1–4), and low (< 1), respectively. Major clusters are shown as group I to XI. **b** Analysis of endogenous *FN1* mRNA expression by conventional (left) and real time RT-PCR assay (right) in OV-shp53 and control cells (Con). The values of endogenous *FN1* mRNA expression detected by conventional RT-PCR assay were normalization to GAPDH. The fold changes in mRNA expression for both assays are presented as means±SDs. The experiment was performed in triplicate. **c** Western blot analysis for the indicated proteins in total lysates from OV-shp53 and control cells (Con). **d** OVISE cells were transfected with *FN1* reporter constructs, together with either p53wt or p53mt. Relative activity was determined based on arbitrary luciferase light units normalized to pRL-TK activity. The activities of the reporter plus the effector relative to that of the reporter plus empty vector are shown as means±SDs. The experiment was performed in duplicate

To identify genes that are differentially expressed following p53-KD, NGS assays were carried out using total RNAs extracted from OV-p53-KD cells. A total of 12,051 and 13,094 genes in OV-shp53#2 and OV-shp53#8 cells were dysregulated, respectively. Of these, 57 and 83 genes were upregulated or downregulated over 5-fold, respectively, in the p53-KD cells as compared to the mock cells. As shown Fig. 5a, hierarchical clustering revealed that the genes could be readily categorized into eleven groups, and we focused on the *FN1* gene in group IV that was overexpressed by 17-fold. *FN1* mRNA and protein expression were apparently increased in OV-p53-KD cells, along with increased expression of integrin β1, β3, and β3 (Fig. 5b, c). Moreover, *FN1* promoter activity was repressed by transfection of p53wt, but not p53mt (Fig. 5d).

**Fig. 6:**
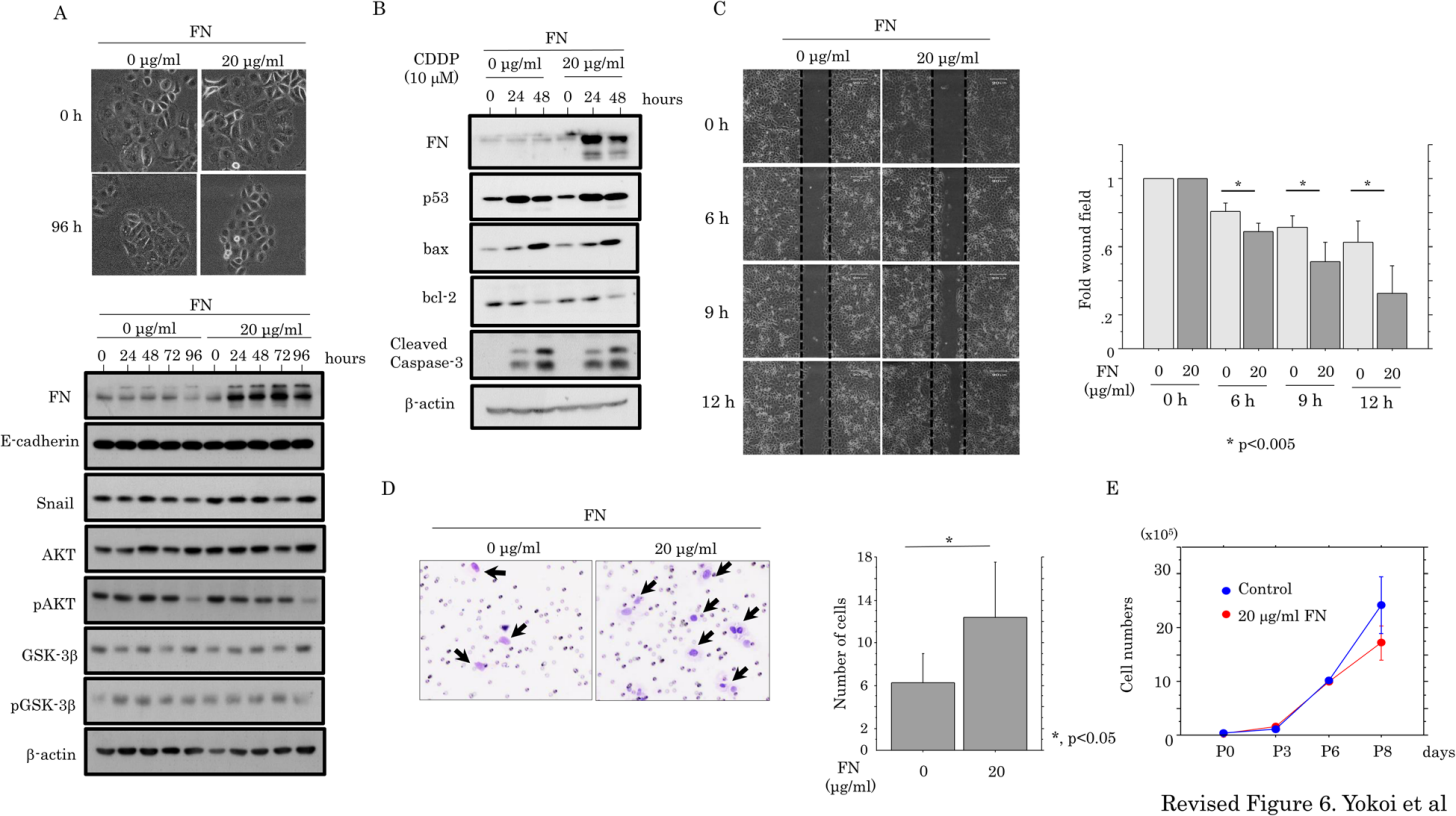
Relationship between FN and cell kinetics. **a** Upper: phase-contrast images of OVISE cells treated with 20 μg/ml FN for 96 h. Note there was no alteration in cell morphology during the treatment. Lower: western blot analysis for the indicated proteins in total lysates from OVISE cells with or without 20 μg/ml FN treatment. **b** Western blot analysis of the indicated proteins in total lysates from OVISE cells with or without 10 μM CDDP treatment in the presence or the absence of 20 μg/ml FN. **c** Left: wound-healing assay with OVISE cells with or without 20 μg/ml FN treatment. A scratch ‘wound’ was made in the middle of cells grown to confluency, and phase contrast images were taken after 6, 9, and 12 h. Right: the values of wound areas in 0 h were set as 1. The fold changes in wound areas are presented as means±SDs. **d** Migration rate measured using the transwell assay. Left: the OVISE cells with or without 20 μg/ml FN treatment were seeded in 24-well transwell plates and incubated for 24 h in medium without serum. The cells were stained by HE and counted using light microscope. Right: numbers of migrated cells are presented as means±SDs. **e** The OVISE cells with or without 20 μg/ml FN treatment were seeded at low density. The cell numbers are presented as means±SDs. P0, P3, P6, and P8 are 0, 3, 6, and 8 days after cell seeding, respectively

Since FN is an EMT-related marker [23, 24], we asked whether there was an association of FN with either EMT or cell motility. OVISE cells treated with FN did not show any changes in cell morphology or expression of E-cadherin, Snail, Akt and GSK-3β (Fig. 6a); the expression of apoptosis-related molecules was also unchanged (Fig. 6b). In contrast, both scratch and migration assays revealed that FN treatment resulted in a significant increase in migration capacity (Fig. 6c,d), along with a decrease in proliferation at later stages (Fig. 6e). These findings suggest that FN is an important determinant of cellular function in p53-KD cells due to its effects on cell mobility and proliferation, rather than via modulation of EMT or apoptosis.

### Prognostic significances of FN and p53 expression in OCCCa/OHGSeCa

**Fig. 7:**
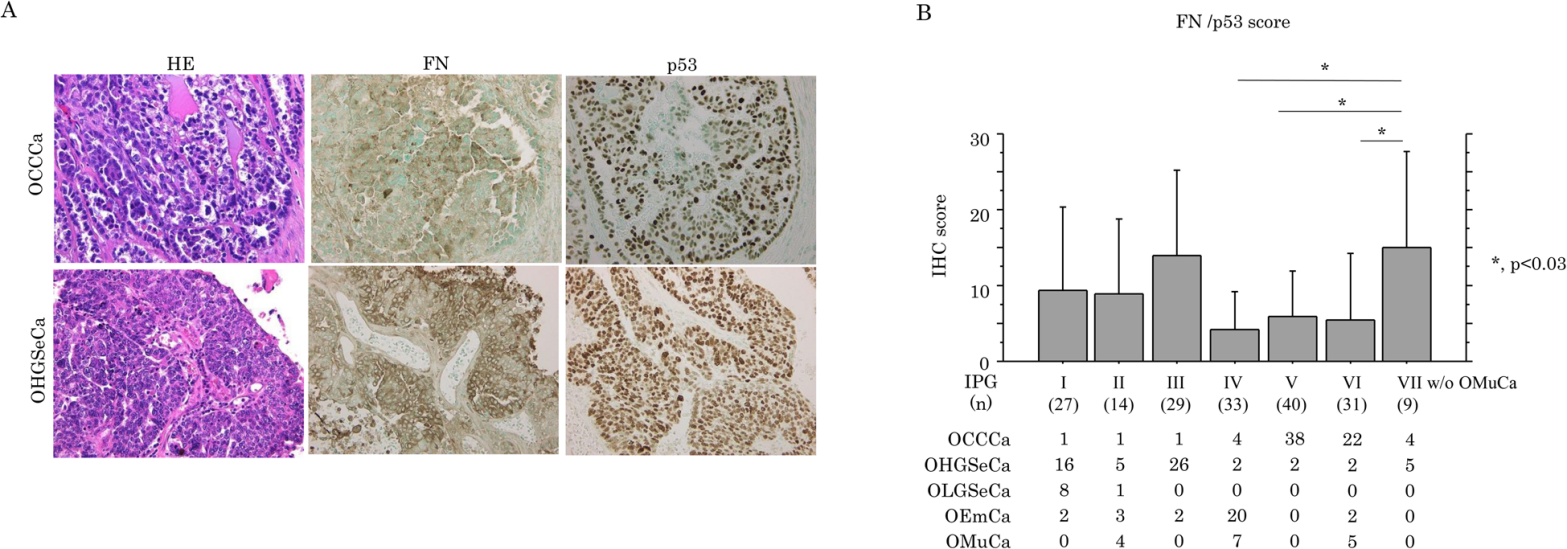
IHC findings in serial sections of OCCCa and OHGSeCa tumors (**a**) Staining by HE and IHC staining for the indicated proteins in OCCCa and OHGSeCa. Original magnification, × 100. (**b**) FN/p53 IHC scores in the immunoprofile groups (IPGs) of OECa including OCCCa, OHGSeCa, OLGSeCa, OEmCa, and OMuCa. OMuCa are excluded from IPG VII (w/o OMuCa). The data shown are as means±SDs

Representative IHC findings for FN and p53 in OCCCa and OHGSeCa are illustrated in Fig. 7a, demonstrating cytoplasmic immunostaining for FN and nuclear staining for p53. FN score was significantly higher in OCCCa as compared to that of OHGSeCa, in contrast to a significantly higher p53 score in the latter (Supplementary Figure S2A). Previously, we used hierarchical clustering analysis to identify seven immunopurified groups (IPGs) in OECa including OCCCa, OHGSeCa, OLGSeCa, OEmCa, and OMuCa [12]. Here, we observed that average FN/p53 scores were significantly higher in IPG VII, which includes OCCCa/OHGSeCa with the HNF-1β+/p53+/ARID1A immunophenotype, and lower in the IPGs IV, V, and VI (Fig. 7b). Similar findings were also observed in p53, but not FN, scores among IPGs including the five OECa histotypes (Supplementary Figure S2B).

The FN scores were also significantly associated with tumor histotype, tumor size, and lymph node metastasis in OCCCa; p53 score was also significantly correlated with clinical stage, histotype, tumor size, and distant metastasis in OHGSeCa (Table 2).

**Fig. 8:**
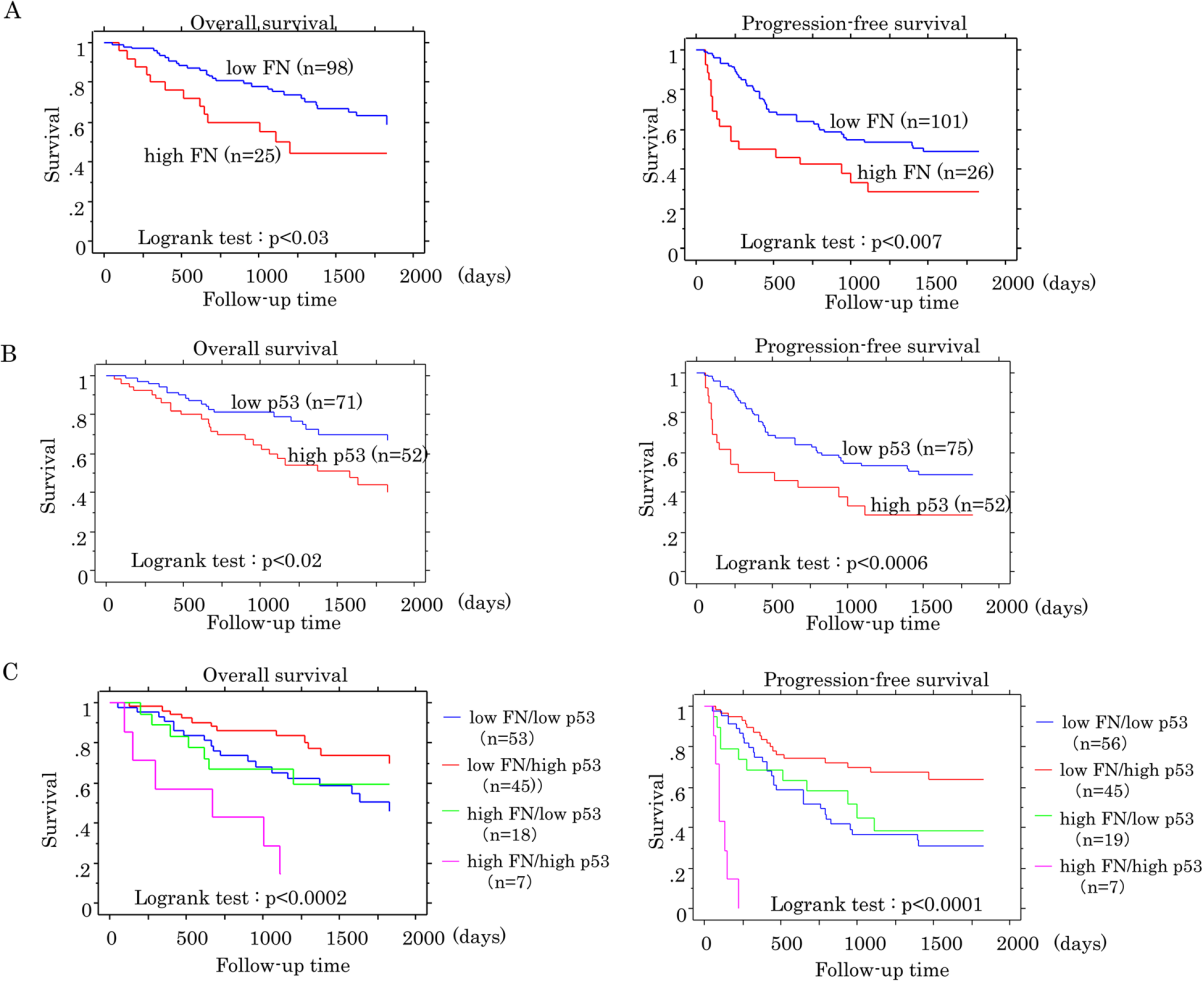
Relationship between FN and p53 expression and prognosis in OCCCa/OHGSeCa. OS (left) and PFS (right) relative to FN (**a**), p53 (**b**), and combined FN and p53 expression (**c**) in OCCCa/OHGSeCa. N, number of cases

The Kaplan-Meier curves showed that patients with high FN and p53 scores had poorer OS and PFS when compared to patients with low scores in the OCCCa/OHGSeCa category (Fig. 8a, b), although such associations were not observed in p53 scores in OHGSeCa (Supplementary Figure S3). Patients with a combination of high FN and high p53 scores also had the worst OS and PFS in OCCCa/OHGSeCa, whereas patients with low values of both scores had the best prognosis (Fig. 8c).

Univariate Cox progression hazards regression revealed that FN, p53, age, tumor histotype, FIGO stage, lymph node metastasis, distant metastasis, and residual tumors after surgical treatment were significant prognostic factors for OS or PFS in OCCCa/OHGSeCa. In addition, multivariate Cox regression analysis showed that FN, FIGO stage, and distant metastasis were significant and independent prognostic factors for OS or PFS (Table 3). These findings suggest that a combined IHC analysis of FN and p53 expression is useful for prognostic prediction of OCCCa/OHGSeCa.

**Table 3:** Univariate and multivariate anayses for overall survival and progression-free survival in OCCCa/OHGSeCa

Univariate analysis					Multivariate analysis				
Variables	Cutt-off	Log rank c2	p-value	factor	Variable	Cut-off	Hazard Ratio	95% CI	p-value
Overall survival					Overall survival				
Fibronectin	2/3	4.9	0.02	High score	Fibronectin	2/3	2.6	1.2–5.4	0.01
p53	5/6	6.4	0.01	High score	p53	5/6	0.9	0.4–2.0	0.8
Histological type	OCCCa/OHGSeCa	11.2	0.0008	OHGSeCa	Histological type	OCCCa/OHGSeCa	0.4	0.2–1.1	0.07
Age	57/58	0.4	0.52		FIGO stage	I/II・III・V	0.2	0.06–0.7	0.01
FIGO stage	I/II・III・V	16.1	< 0.0001	II・III・IV	LN metastasis	−/+	0.8	0.4–1.5	0.5
Tumor size	11.1/11.2	1.1	0.3		Distant metastasis	−/+	0.7	0.3–1.6	0.4
LN metastasis	−/+	7.8	0.005	+	Residual tumor	Present/absent	0.6	0.3–1.2	0.2
Distant metastasis	−/+	8.9	0.002	+	after surgery				
Residual tumor	Present/absent	8.1	0.004	Present					
after surgery									
Progression-free survival					Progression-free survival				
Fibronectin	2/3	7.6	0.006	High score	Fibronectin	2/3	3.3	1.7–6.4	0.0005
p53	5/6	12.2	0.0005	High score	p53	5/6	1.3	0.6–2.8	0.6
Histological type	OCCCa/OHGSeCa	16.6	< 0.0001	OHSeGCa	Histological type	OCCCa/OHGSeCa	0.6	0.3–1.3	0.2
Age	57/58	3.9	0.04	58≦	Age	57/58	0.8	0.4–1.3	0.3
FIGO stage	I/II・III・V	32.7	< 0.0001	II・III・IV	FIGO stage	I/II・III・V	0.2	0.06–0.5	0.001
Tumor size	11.1/11.2	0.7	0.3		LN metastasis	−/+	0.7	0.4–1.2	0.2
LN metastasis	−/+	14.5	0.0001	+	Distant metastasis	−/+	0.5	0.2–0.9	0.03
Distant metastasis	−/+	22.1	< 0.0001	+	Residual tumor	Present/absent	0.7	0.4–1.3	0.3
Residual tumors	Present/absent	10	0.001	Present	after surgery				
after surgery									

*LN* lymph node, *n* number of cases

## Discussion

Although extensive studies on the gain-of-function (GOF) effects of p53mt have been conducted using in vitro cell culture systems, several in vivo models have indicated that the primary effect of p53 mutation is the loss of p53wt function, with little or no GOF effect on tumorigenesis. Thus, the GOF of a particularly p53mt is likely to be determined by tissue- and tumor type-specific factors. For example, MMTV-*HrasTP53*^*R172H/R172H*^ and MMTV-*Hras/TP53*^*−/−*^ mice were very similar with regard to age of salivary tumor onset, tumor growth rate, tumor histopathological features, and response to a DNA-damaging agent [25]. This was also the case in a *K-ras*-driven lung cancer model, as well as in the context of *WAP-Cre*-induced expression of the p53R270H mutant in p53-null-mouse mammary glands, and in p53R172H homozygous knock-in mouse models [26–28]. In addition, the GOF activity of any particular p53mt is largely dependent on multiple signals required for its post-translational stabilization: the presence of such signals is likely to vary among both normal and tumor cells [29]. Based on the above evidence, we examined alterations in the p53 signaling pathway using cells following shRNA-mediated knockdown of p53wt.

Here, we provide clear evidence that p53 loss leads to upregulation of HNF-1β, as well as ARID1A, at both mRNA and protein levels in OCCCa cells. Moreover, transfection of p53wt, but not p53mt, represses *HNF-1β* promoter activity, suggesting that alterations in the *p53* gene may play an important role in development of OCCCa that have the HNF-1β+/p53+ /ARID1A+ immunophenotype. However, analysis of the TCGA database revealed that expression of *HNF1β* and *ARID1A* mRNA was not correlated with p53 status in OHGSeCa. Given the evidence that HNF-1β is a sensitive and specific marker for OCCCa and is not expressed in OHGSeCa with clear cell changes [30], it appears that some cell type-specific factors may also be required for establishment of OHGSeCa with the immunophenotypic features we specify above.

We also found that p53-KD cells have dramatically altered cell morphology and are more fibroblast-like in appearance; these changes are accompanied by increased expression of E-cadherin-repressor Snail, as well as pAkt and pGSK-3β, and decreased E-cadherin expression. Although *Snail* promoter activity was specifically repressed by p53wt, we did not observe differences in *Snail* mRNA levels between p53-KD and the mock-transfected cells. In general, Snail expression is decreased through GSK-3β-mediated phosphorylation/degradation [31], while GSK-3β activity is inhibited following activation of Akt [32]. Given the mutual antagonism between the p53 and Akt networks [33–35], it is suggested that loss of p53 function leads to post-translational upregulation of Snail through activation of the Akt/GSK-3β axis, which in turn leads to induction of EMT. Interestingly, p53mt can directly bind and trans-repress the promoter of *miR-130b*, a microRNA that specifically downregulates ZEB1, leading to the upregulation of BMI-1 and Snail [36]. In addition, p53wt can induce MDM2 mediated degradation of Snail [37].

Here, we found that p53-KD cells had a reduced proliferative rate and enhanced migration capability, along with enhanced G2/M arrest and increased p27^kip1^ expression. Moreover, cyclin B1 expression also decreased progressively after serum stimulation in p53-KD cells. Our findings are consistent with those of previous reports. First, p27 ^kip1^ is important for the initial activation of G2/M checkpoint in response to low-dose ionizing radiation [38], in line with evidence that Cdc2, which is essential for entry into mitosis, physically interacts with, and is inhibited by p27 ^kip1^ [39, 40]. In addition, binding of Cdc2 to cyclin B1 is required for its activity and repression of cyclin B1 contributes to blocking entry into mitosis. Second, hematopoietic cells expressing p53wt arrest in both G1 and G2 phase of the cell cycle, while p53-null cells or cells overexpressing p53mt exhibited only G2 arrest [41]. Third, reduced levels of p53 correlate with increased G2/M phase arrest in response to paclitaxel treatment in normal human fibroblast depleted of functional p53 [42]. Finally, migratory cells have a lower proliferation rate in comparison with cells in the tumor core, indicating an inverse correlation between cell proliferation and mobility [43–45].

Our findings also revealed that susceptibility to apoptosis in response to CDDP treatment was significantly inhibited in p53-KD cells. This may be explained by the prolonged high levels of pAkt in response to loss of p53 function, because there is an ‘all-or-none’ switching behavior between a pro-survival cellular state (low p53 and high Akt levels) and a pro-apoptotic state (high p53 and low Akt levels) [33], as well as decreased expression of the pro-apoptotic protein, bax [46].

An important finding in this study was that *FN1* mRNA and protein expression were significantly increased in p53-KD cells, while *FN1* promoter activity was repressed by p53wt, but not p53mt, in line with other studies [47, 48]. Moreover, treatment of OCCCa cells with FN resulted in an enhanced migration capability and a reduced proliferation rate. In contrast, the effects of FN on cell morphology, expression of EMT-related molecules, and susceptibility to apoptosis were minimal. Taken together with our results that p53-KD increases the expression of the FN receptors integrins β1, β2, and β3 [49], we conclude that FN upregulation due to loss of p53 function is closely associated with enhancement of cell mobility, but not induction of EMT and apoptotic features. However, we could not demonstrate a direct correlation between FN and p53 scores in the OCCCa/OHGSeCa category, indicating that a p53-independent pathway must regulate FN expression in the tumors, particularly in OCCCa. In fact, FN expression is upregulated through the PI3K/Akt pathway in tamoxifen-resistant breast cancer cells [50].

Finally, patients with a combination of high FN and high p53 IHC scores had significantly worse OS and PFS than did patients with low values for both scores in OCCCa/OHGSeCa. Both FN and p53 scores were also significantly associated with several unfavorable clinicopathological factors in the tumors. Moreover, multivariate Cox regression analysis also showed that FN, but not p53, was a significant and independent unfavorable prognostic factor for OS and PFS. Furthermore, there was a positive association between high FN score, enlarged tumor size and nodal metastasis, suggesting that combined IHC analysis for FN and p53 expression may have great utility in OCCCa/OHGSeCa prediction and prognosis.

## Conclusion

**Fig. 9:**
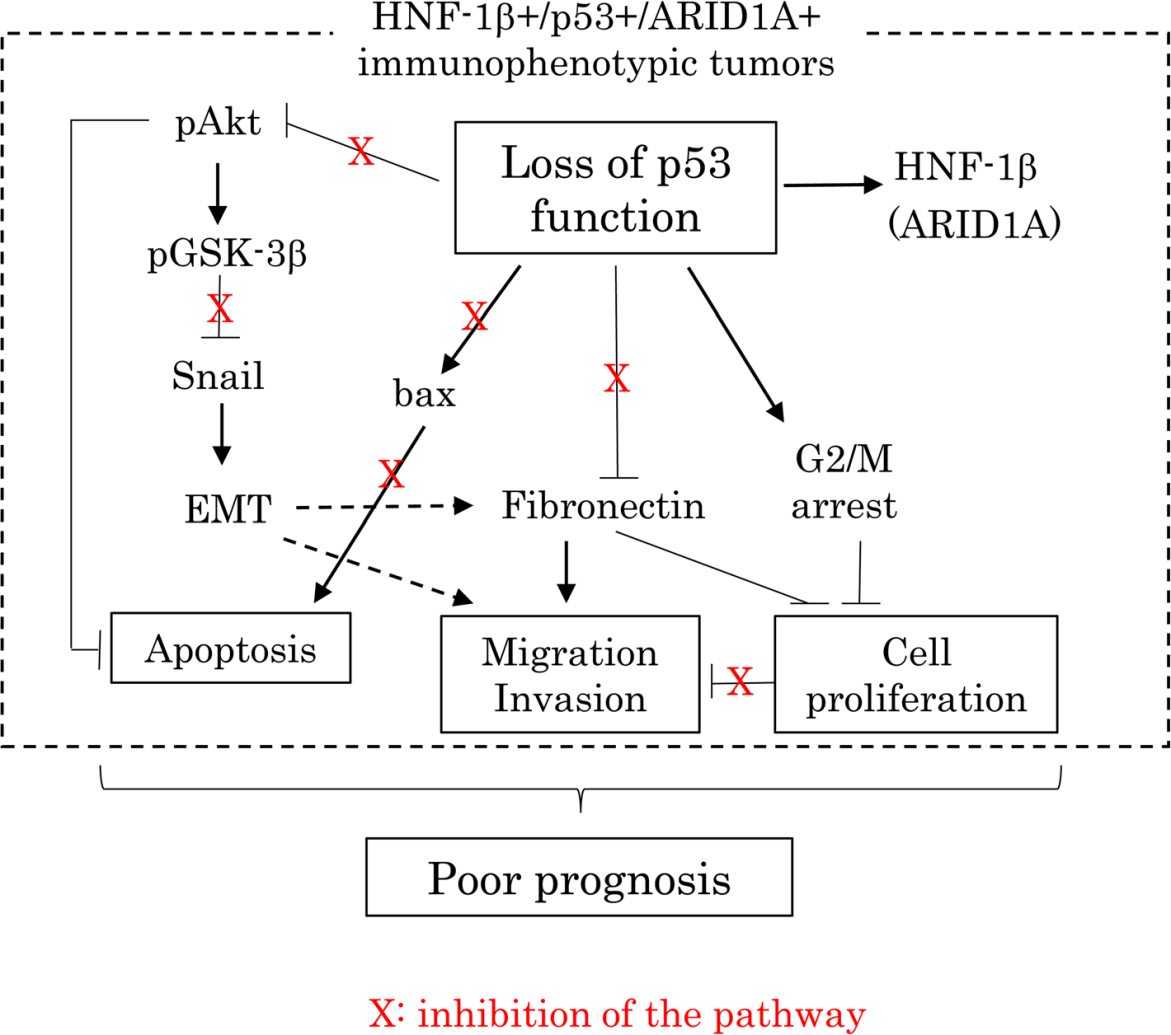
Schematic representation of the interplay between the p53 pathway, FN expression, and the Akt/GSK-3β/Snail axis in the aggressive OCCCa/OHGSeCa

Upregulation of FN following loss of p53 function may influence the malignant properties of OCCCa/OHGSeCa, particularly in those tumors with an HNF-1β+/p53+/ARID1A+ immunophenotype. The accompanying induction of EMT/CSC properties and inhibition of apoptosis due to p53 abnormalities also contribute to the establishment and maintenance of tumor phenotypic characteristics (Fig. 9).

## Supplementary information

**Additional file 1 : Supplementary Figure S1** p53, HNF-1β, and ARID1A expression in OCCCa cells. (A) Western blot analysis for the indicated proteins in total lysates from four OCCCa cell lines. Note p53 mutation was only presented in ES-2 cells. wt, wild-type. (B) Analysis of TCGA data for associations between *p53* gene abnormalities with expression of *HNF-1β* and *ARID1A* mRNAs (left and right, respectively).

**Additional file 2 : Supplementary Figure S2** FN and p53 expression in OECa. (A) FN and p53 scores in OECa. (B) FN/p53 IHC scores in the immunoprofile groups (IPGs) of OECa including OCCCa, OHGSeCa, OLGSeCa, OEmCa, and OMuCa. OMuCa are excluded from IPG VII (w/o OMuCa). The data shown are as means±SDs.

**Additional file 3 : Supplementary Figure S3** Relationship between FN and p53 expression and prognosis in OCCCa or OHGSeCa. (A) OS (left) and PFS (right) relative to FN and p53 expression (upper and lower, respectively) in OCCCa. B) OS (left) and PFS (right) relative to FN and p53 expression (upper and lower, respectively) in OHGSeCa. N, number of cases.

## Data Availability

Data and materials will be shared.

## Abbreviations

OECa: Ovarian epithelial carcinomas
OHGSeCa: Ovarian high grade serous carcinoma
OCCCa: Ovarian clear cell carcinoma
KD: Knockdown
p53wt: Wild-type p53
p53mt: Mutant type p53
IHC: Immunohistochemistry
EMT: Epithelial-mesenchymal transition
FN: Fibronectin
OS: Overall survival
PFS: Progression-free survival
IPG: Immunoprofile group
