# Supplementary figures and images for: Upregulation of fibronectin following loss of p53 function is a poor prognostic factor in ovarian carcinoma with a unique immunophenotype
Source: Cell Commun Signal. 2020 Jul 7;18:103. doi: 10.1186/s12964-020-00580-3 (PMC7341596; doi:10.1186/s12964-020-00580-3)

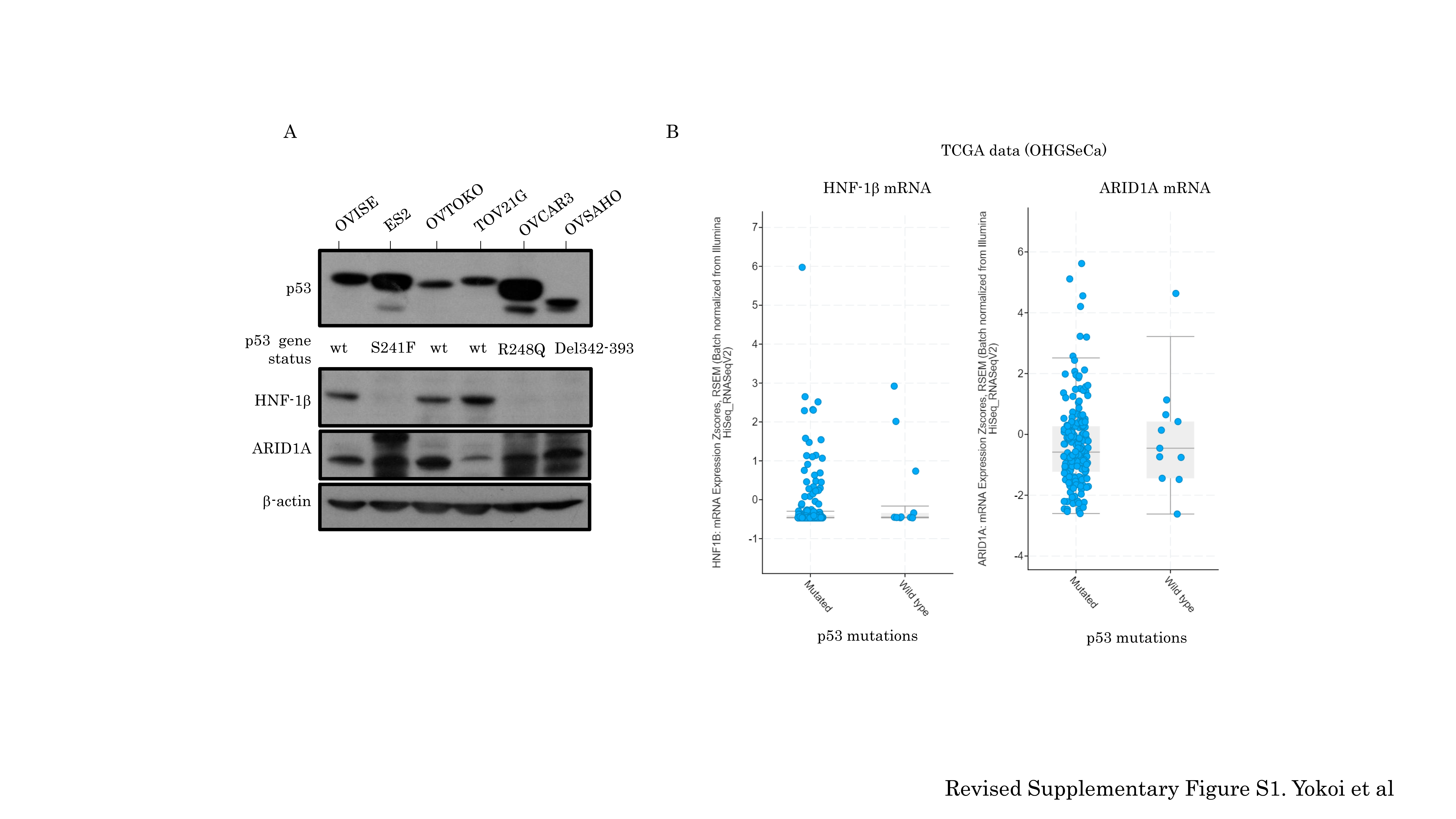

Supplement: Supplementary file 2 — Additional file 1 : Supplementary Figure S1 p53, HNF-1β, and ARID1A expression in OCCCa cells. (A) Western blot analysis for the indicated proteins in total lysates from four OCCCa cell lines. Note p53 mutation was only presented in ES-2 cells. wt, wild-type. (B) Analysis of TCGA data for associations between p53 gene abnormalities with expression of HNF-1β and ARID1A mRNAs (left and right, respectively). [file 12964_2020_580_MOESM2_ESM.tif]

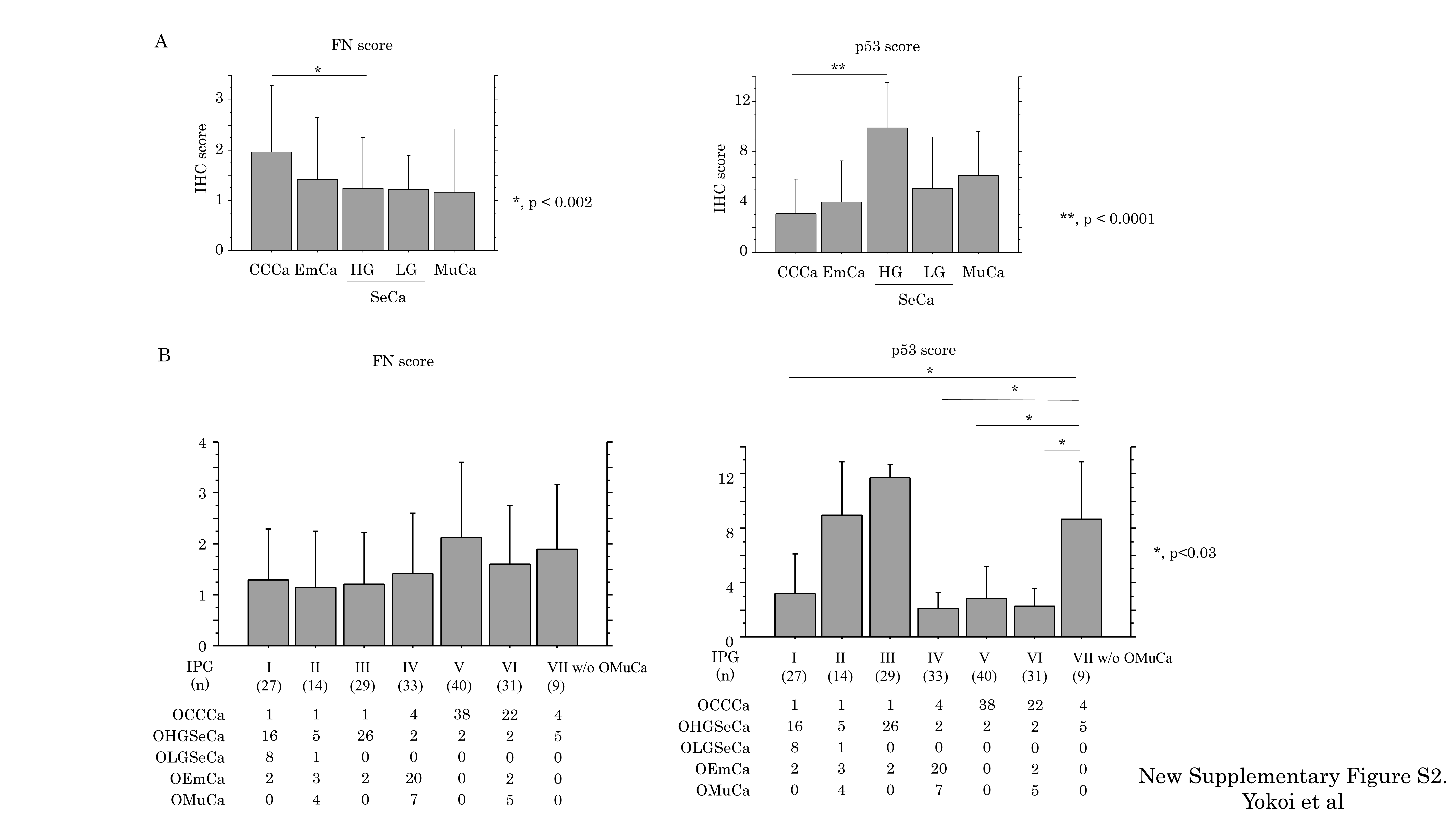

Supplement: Supplementary file 3 — Additional file 2 : Supplementary Figure S2 FN and p53 expression in OECa. (A) FN and p53 scores in OECa. (B) FN/p53 IHC scores in the immunoprofile groups (IPGs) of OECa including OCCCa, OHGSeCa, OLGSeCa, OEmCa, and OMuCa. OMuCa are excluded from IPG VII (w/o OMuCa). The data shown are as means±SDs. [file 12964_2020_580_MOESM3_ESM.tif]

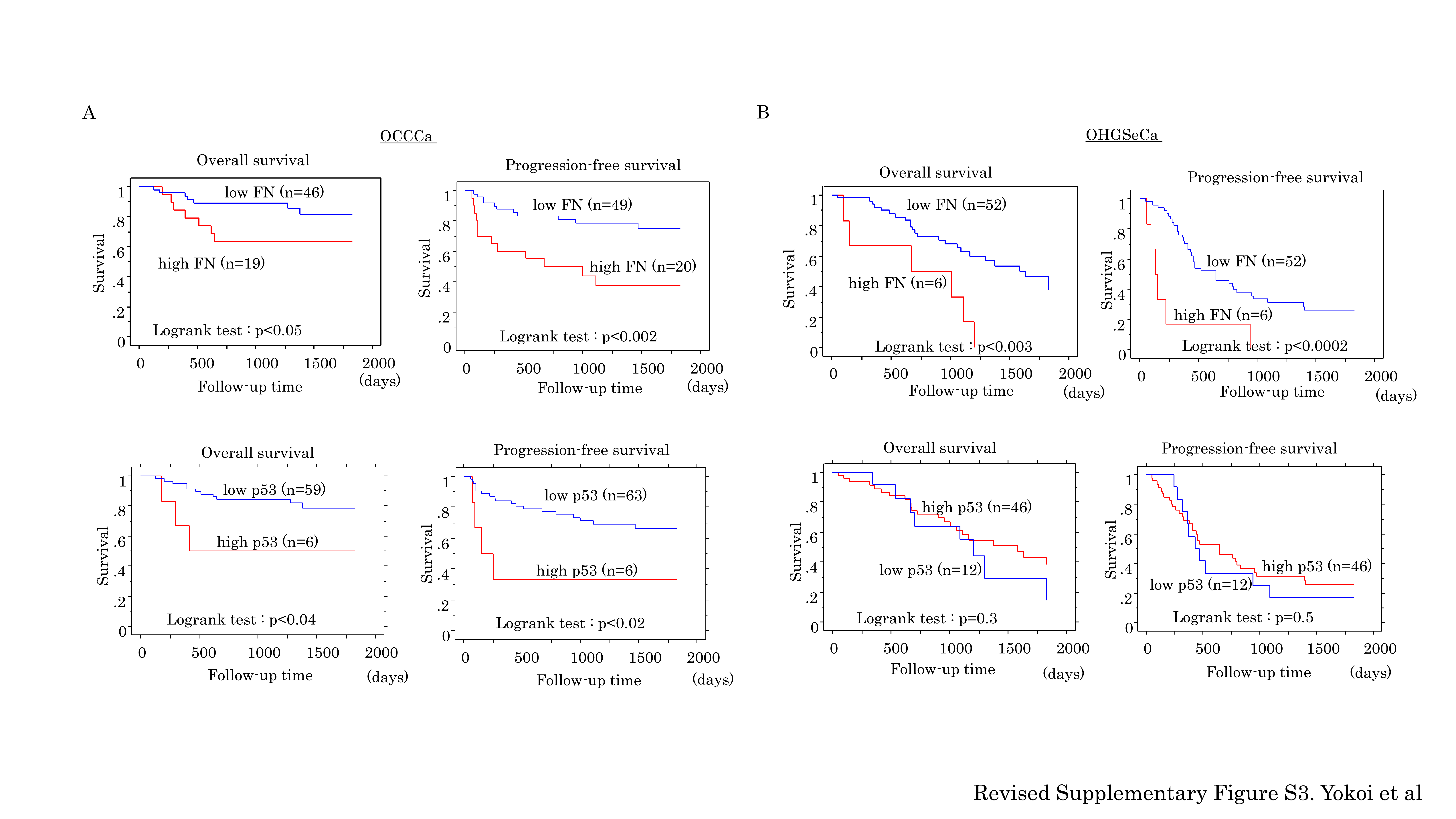

Supplement: Supplementary file 4 — Additional file 3 : Supplementary Figure S3 Relationship between FN and p53 expression and prognosis in OCCCa or OHGSeCa. (A) OS (left) and PFS (right) relative to FN and p53 expression (upper and lower, respectively) in OCCCa. B) OS (left) and PFS (right) relative to FN and p53 expression (upper and lower, respectively) in OHGSeCa. N, number of cases. [file 12964_2020_580_MOESM4_ESM.tif]
